# Clinical and hormonal findings in patients presenting with high IGF-1 and growth hormone suppression after oral glucose load: a retrospective cohort study

**DOI:** 10.1530/EJE-21-0024

**Published:** 2021-06-03

**Authors:** Giulia Carosi, Alessandra Mangone, Elisa Sala, Giulia Del Sindaco, Roberta Mungari, Arianna Cremaschi, Emanuele Ferrante, Maura Arosio, Giovanna Mantovani

**Affiliations:** 1 Endocrinology Unit, Fondazione IRCCS Cà Granda Ospedale Maggiore Policlinico, Milan, Italy; 2 Department of Experimental Medicine, Sapienza University of Rome, Rome, Italy; 3 Department of Clinical Sciences and Community Health, University of Milan, Milan, Italy

## Abstract

**Objective:**

High insulin-like growth factor 1 (IGF-1) and unsuppressed growth hormone (GH) levels after glucose load confirm the diagnosis of acromegaly. Management of patients with conflicting results could be challenging. Our aim was to evaluate the clinical and hormonal evolution over a long follow-up in patients with high IGF-1 but normal GH nadir (GHn < 0.4 μg/L according to the latest guidelines).

**Design:**

Retrospective cohort study.

**Methods:**

We enrolled 53 patients presenting high IGF-1 and GHn < 0.4 μg/L, assessed because of clinical suspicion of acromegaly or in other endocrinological contexts (e.g. pituitary incidentaloma). Clinical and hormonal data collected at the first and last visit were analyzed.

**Results:**

At the first evaluation, the mean age was 54.1 ± 15.4 years, 34/53 were females, median IGF-1 and GHn were +3.1 SDS and 0.06 μg/L, respectively. In the whole group, over a median time of 6 years, IGF-1 and GHn levels did not significantly change (IGF-1 mean of differences: −0.58, *P* = 0.15; GHn +0.03, *P* = 0.29). In patients with clinical features of acromegaly, the prevalence of acromegalic comorbidities was higher than in the others (median of 3 vs 1 comorbidities per patient, *P* = 0.005), especially malignancies (36% vs 6%, *P* = 0.03), and the clinical worsening overtime was more pronounced (4 vs 1 comorbidities at the last visit).

**Conclusions:**

In patients presenting high IGF-1 but GHn < 0.4 μg/L, a hormonal progression is improbable, likely excluding classical acromegaly in its early stage. However, despite persistently low GH nadir values, patients with acromegalic features present more acromegalic comorbidities whose rate increases over time. Close clinical surveillance of this group is advised.

## Introduction

Acromegaly is a rare disease resulting from chronic exposure to high levels of growth hormone (GH) and its main mediator, insulin-like growth factor 1 (IGF-1). Almost all patients have a GH-secreting pituitary adenoma which is a macroadenoma in about 70% (1, 2, 3). Increased morbidity and mortality are well documented in patients with active disease, mainly due to cardiovascular, neoplastic, respiratory, and metabolic comorbidities (3, 4, 5, 6). Therefore, a prompt therapeutic approach is mandatory.

The evaluation of IGF-1 is recommended as a screening test for acromegaly. In the presence of high IGF-1, the diagnosis has to be confirmed by an inadequate GH suppression after an oral glucose tolerance test (OGTT). Diagnostic criteria changed over time as more sensitive assays for GH measurement have become widely available, leading to lower GH nadir (GHn) reference values, up to 0.4 μg/L (2). OGTT is generally diagnostic in the majority of patients. However, some series reported acromegalic patients with adequate GH suppression (7, 8, 9, 10, 11, 12, 13).

In 2002, Barkan and co-workers introduced the term 'micromegaly', describing a group of patients with classical acromegalic features, high IGF-1 but normal mean GH and GHn (14). Since then, other studies reported similar patients, according to various GHn reference values (7, 8, 9, 10, 11, 12, 13), ranging from 2 to 0.4 μg/L. Interestingly, a pituitary adenoma was not identified in all cases, leading to speculation on the disease’s pathophysiology (i.e. enhanced GH receptor activity), but many data on the clinical significance of this condition are still lacking. Moreover, a systematic clinical and hormonal assessment of these patients and data on the possible disease progression over time are not yet available. Therefore, the management remains challenging.

Besides the suspicion of acromegaly, IGF-1 levels are often measured in other clinical contexts, as in the presence of incidentally discovered pituitary neoplasms (15). We know that IGF-1 assessment harbors some limitations such as the lack of adequate age-adjusted normative data and susceptibility to interference from IGF-binding proteins (IGFBP) (16). Thus, clinicians frequently face unexpected high values despite no signs of GH hypersecretion. An apparent low incidence of acromegalic features and comorbidities seems to confirm the absence of disease in this setting (9).

The main aim of the present study was to characterize a large group of patients presenting with GH/IGF-1 discrepancy (high IGF-1 levels but suppressed GH values according to the most recent guidelines) (2), both with and without an initial suspicion of acromegaly. We focused our attention on representative comorbidities of acromegaly and GH/IGF-1 hormonal assessment, and we evaluated the evolution of all cited parameters over a long follow-up period to identify the best management of these patients.

## Subjects and methods

### Patients

We analyzed the data of a group of adult patients referred to a single tertiary Endocrinology Unit (Fondazione IRCCS Ca’ Granda Ospedale Maggiore Policlinico, Milan), between 2008 and 2019, presenting with IGF-1/GH discrepancy (high IGF-1 in at least two occasions in the presence of an adequate GH nadir). A GH nadir of 0.4 μg/L was considered as the reference cut-off according to recent data and guidelines (2, 17, 18). We included patients who had been hormonally evaluated both for suspected acromegaly and in other clinical contexts (e.g. pituitary neoplasms). All conditions which may alter the GH-IGF-1 axis such as pregnancy, hepatic and renal failure, chronic inflammation, malnutrition, or oral estrogen therapy (19, 20, 21) are represented as exclusion criteria.

### Study design and methods

We used medical records to retrospectively and longitudinally investigate data collected at the time of diagnosis (first finding of a discrepancy) and at the last available visit.

Hormonal data included IGF-1, GHn, and GH random levels (GHr). GHr was assessed fasting in the morning, before starting OGTT. GHn was defined as the lowest GH value at any time during 2-hour OGTT (blood samples for GH were collected at 30, 60, 90, 120 min after glucose load).

The presence of acromegalic signs and symptoms (acromegalic facies, acral enlargement, headache, paraesthesia, hyperhidrosis, and arthralgia) were collected along with the diagnosis of typical comorbidities including goiter, arterial blood hypertension, cardiopathy (counting history of congestive heart failure, evidence of ventricular hypertrophy, diastolic/systolic dysfunction, or mitral/aortic abnormalities on echocardiography), colonic polyps, malignant neoplasms, carpal tunnel, and glucose metabolism alterations (GMA) (22, 23, 24, 25, 26, 27). According to the international criteria, we considered in GMA impaired fasting glucose (IFG), impaired glucose tolerance (IGT), and diabetes mellitus (DM) (2, 28). Insulin resistance has been determined by HOMA index calculation (HOMA-IR) according to the formula: fasting insulin (mU/L) × fasting glucose (nmol/L)/22.5 (29). We also included BMI as an anthropometric parameter (weight in kilograms divided by the square of height in meters).

The suspicion of acromegaly, based on the presence of acromegalic features (physical changes including the prominence of brow, prognathism, macroglossia, acral overgrowth, nose, and lips enlargement), was determined by three board-certified experts in endocrinology with at least 5 years of clinical practice in this field.

In addition, we included the results of MRI of the sellar region before and after gadolinium contrast. We registered the presence of pituitary adenomas, empty sella, and other nonspecific alterations such as pituitary stalk deviation or sellar floor depression.

To better characterize patients with 'micromegaly', as defined by previous authors (9, 11, 14), patients with typical acromegalic features (group 1, GR1) were separately analyzed and compared with the others (group 2, GR2).

The Local Ethical Committee (Comitato Etico Milano Area 2) approved this protocol study.

### Assays

GH was assayed by a chemiluminescence method (Immulite 2000, Siemens Medical Solutions Diagnostics, detection limit of 0.01  μg/L). Standards used for calibration were IS 80/505 from 2008 to July 2010 and IS 98/574 from August 2010. IGF-1 levels were measured by a chemiluminescent immunometric assay (Immulite 2000 IGF-1; Siemens Medical Solutions Diagnostics), and standards used for calibration were IRR 87/518 from 2008 to April 2017 and IS 02/254 from May 2017. IGF-1 values were expressed in standard deviation scores (SDS). We obtained SDS values accordingly to the methods provided by Chanson and colleagues (30).

### Statistical analysis

We described continuous parameters with normal distribution as mean ± s.d. and non-Gaussian distributions as median with interquartile range (IQR). Continuous and non-Gaussian data were compared using *t*-test and Wilcoxon–Mann–Whitney test, respectively. Categorical data were presented as percentage (%), proportion (/) and analyzed using the Chi-squared test or Fisher's exact test if the expected value was <5. For statistical calculation, GH values < 0.05 μg/L were arbitrarily set to 0.04 μg/L. To investigate the association of GH levels with the number of comorbidities, Spearman’s correlation analysis was conducted. One-way ANOVA test for linear trend was used to compare GHn levels among five groups sub-divided based on comorbidities’ number. ROC analysis assessed threshold values of GHn that detect patients with numerous comorbidities. The optimum sensitivity and specificity were determined using the Youden index (J). *P*-values < 0.05 were considered statistically significant. All statistical analyses were performed using SPSS, version 24 (IBM).

## Results

### Hormonal and clinical data of the entire population

We selected 53 patients, 64.2% females and 35.8% males, with an average age at diagnosis of 54.1 ± 15.4 years (range: 20–80). Twenty-four females (70.6%) were post-menopausal. Mean BMI was 27 ± 4.3 kg/m^2^ and HOMA-IR was 2.2 ± 1.2.

In 25/53 patients (47.2%), IGF-1 levels were evaluated because of clinical suspicion of acromegaly. The other 28 patients (52.8%) had IGF-1 measured for a variety of reasons, including seven pituitary adenomas (5 pituitary incidentalomas, macroadenomas conditioning neuro-ophthalmological symptoms), 11 suspected hypopituitarism (4 hypogonadism, 3 hypothyroidism, 2 GH-deficiency, 1 diabetes insipidus, 1 hypoadrenalism), three empty sella, one suspected Cushing disease, one sellar meningioma, one hyperprolactinemia, four other endocrinopathies (1 hyperandrogenism, 1 MEN-1, 1 adrenal tumor, 1 thyroid cancer). Hypopituitarism was excluded in all cases except for two patients with hypogonadism.

At diagnosis, IGF-1 in the whole group was +3.1 SDS (+2.5 to +4.0), median GHr 1.1 μg/L (0.18–2.5) and GHn 0.06 μg/L (0.05–0.16). GHr and GHn levels were higher in females (F) than in males (M): GHr (F) = 1.7 μg/L (0.9–3.2), (M) = 0.15 μg/L (0.05–0.76),* P* <0.0001; GHn (F) = 0.1 μg/L (0.05–0.2), (M) = 0.05 (0.04–0.09),* P =* 0.04, as expected (31).

Taken as a whole group, typical symptoms of acromegaly were distributed as follows: arthralgia in 28.3%, paraesthesia in 24.5%, headache in 20.8%, and hyperhidrosis in 9.4% of subjects. Acral enlargement was reported in 50.9%. The median number of comorbidities per patient was 2 (IQR: 1–3.5). Among acromegalic comorbidities, we registered glucose metabolism alterations in 36/53 (67.9%) patients (13 IFG, 20 IGT, and 13 DM), hypertension in 43.4%, goiter in 35.8%, cardiopathy in 26.4%, carpal tunnel in 18.9%, malignant neoplasms in 18.9 %, and colonic polyps in 18.9%. We underlie that only 15 colonoscopies were performed, leading to a possible overall underestimation of polyps. Neoplasms were represented by medullar thyroid cancer (2/10), neuroendocrine gastrointestinal tumor (2/10), uterine (2/10), colorectal (1/10), kidney (1/10), breast (1/10), and ovary (1/10) cancer.

Neuroimaging was available in 45/53 (84.9%) patients. The overall number of pituitary adenomas was 21/45 (46.6%, 4 = macro and 17 = microadenomas). Empty sella was present in 8/45 (17.8%), nonspecific alterations in 12/45 (26.7%), and a completely normal finding was reported in 9/45 (20%).

### Hormonal and clinical data of patients with and without acromegalic features

Patients with acromegalic features (GR1) appeared clinically and hormonally different from the others (GR2), showing more comorbidities, more symptoms, and higher GHn values (details in Table 1). Acral enlargement was reported in all patients of GR1 according to inclusion criteria and in 2/28 of GR2 (*P* < 0.0001). Considering that some comorbidities are expected to increase with aging, we compared data on comorbidities in patients of similar age (we excluded 11 patients younger than 45 years, who all belonged to GR2).

**Table 1:** Clinical and hormonal features of patients with and without acromegalic features at the time of diagnosis of GH/IGF-1 discrepancy. IGF-1 values did not significantly differ between the two groups while patients with acromegalic features (GR1) displayed higher GH nadir levels and more comorbidities. Data are presented as median and IQRs.

Parameters	GR1	GR2	*P*-value
Cases, *n*	25	28	
Sex, F/M (%)	20/5 (80)	14/14 (50)	0.02*
Age at diagnosis, years	59 (56.5–67.5)	52 (31.5–60.5)	<0.001*
BMI, kg/m^2^	27.4 ± 5	27.4 ± 3.2	0.1
HOMA-IR	2.0 ± 0.9	2.3 ± 1	0.5
Hormonal values			
IGF-1, +SDS	3.15 (2.7–4.5)	3.1 (2.5–4)	0.57
GHn, μg/L	0.12 (0.06–0.26)	0.05 (0.04–0.08)	0.002*
GHn (F menop), μg/L	0.12 (0.07–0.23)	0.06 (0.04–0.06)	0.04*
GHn (F pre-menop), μg/L	–	0.12 (0.05–0.24)	–
GHn (M), μg/L	0.16 (0.06–0.32)	0.05 (0.04–0.06)	0.033*
GHr, μg/L	1.53 (0.42–2.74)	0.76 (0.12–2.3)	0.1
GHr (F menop), μg/L	1.83 (1.41–4.9)	1.19 (0.96–2.9)	0.29
GHr (F pre-menop), μg/L	–	1.57 (0.23–4.3)	–
GHr (M), μg/L	0.20 (0.08–0.46)	0.13 (0.05–1.18)	0.36
Acromegaly signs and symptoms			
Acral enlargement, *n* (%)	25 (100)	2 (7.1)	<0.0001*
Paraesthesia, *n* (%)	10 (40)	3 (10.7)	0.02*
Arthralgia, *n* (%)	10 (40)	5 (18)	0.12
Headache, *n* (%)	7 (28)	4 (14.3)	0.34
Hyperhidrosis, *n* (%)	3 (12)	2 (7.1)	0.67
Comorbidities (age > 45 years)			
Cases (>45 years), *n*	25	17	
Age, years	59 (56.6–67.5)	55 (52.5–69.5)	0.26
Comorbidities per patient, *n*	3 (2–5)	1 (0–2)	0.005*
Goiter, *n* (%)	13 (52)	5 (29)	0.21
Carpal tunnel, *n* (%)	9 (36)	1 (6)	0.031*
Colonic polyps, *n* (%)	6 (24)	3 (18)	0.72
Malignancies, *n* (%)	9 (36)	1 (6)	0.031*
Hypertension, *n* (%)	15 (60)	7 (41)	0.35
Cardiopathy, *n* (%)	9 (36)	5 (29)	0.75
DM, *n* (%)	9 (36)	4 (24)	0.51
Pituitary MRI findings			
MRI performed, *n*	21	24	
Macro, *n* (%)	2 (9.5)	2 (8.3)	>0.99
Micro, *n* (%)	7 (33.3)	10 (41.7)	0.76
Empty sella, *n* (%)	2 (9.5)	6 (25)	0.25

DM, diabetes mellitus; F, female; GHn, GH nadir; GHr, GH random; GR1, group 1; GR2, group 2; M, male; macro, macroadenoma; menop, menopausal; micro, microadenoma; n, number; pre-menop, pre-menopausal. *statistically significant

MRI scan was available in 45/53 (21/25 of GR1, 24/28 of GR2). In GR1, MRI was performed because of the suspicion of acromegaly and revealed a pituitary adenoma in 9/21, empty sella in 2/21, nonspecific signs in 6/21, and normal findings in 4/21. In GR1, the tumor mean diameter was 4.9 ± 2.2 mm, in GR2 9.1 ± 5.6 mm,* P =* 0.073.

### Relationship between assessed parameters and comorbidities

**Figure 1:**
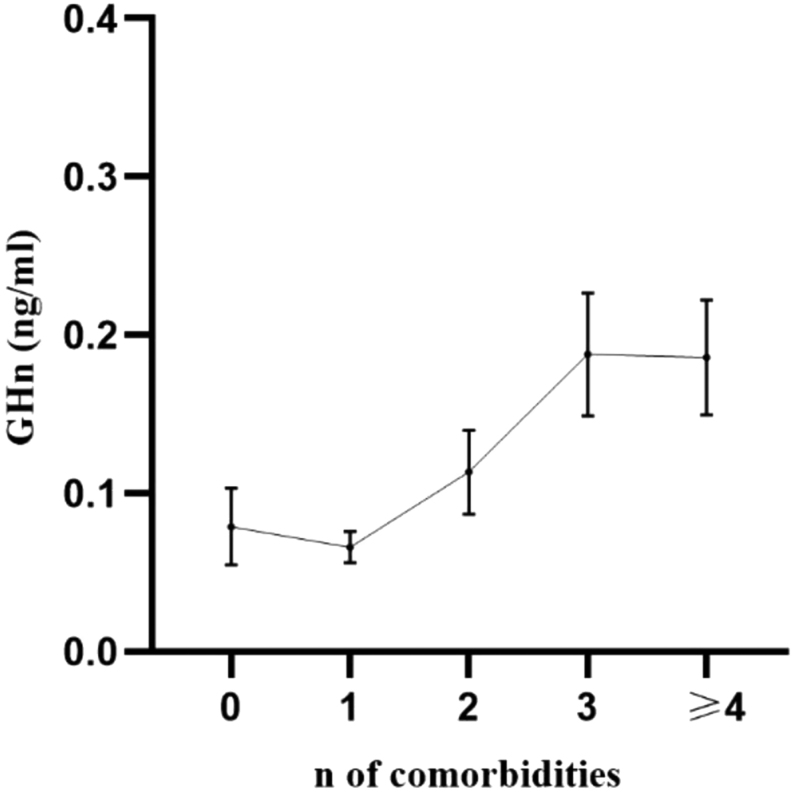
GH nadir shows a significant trend (F = 2.849, *P* = 0.035) to increase according to the number of comorbidities per patient, even if GH nadir is lower than 0.4 ng/mL. Data are expressed as mean ± s.e.m. GHn, GH nadir; n, number.

Spearman’s rank correlation analysis revealed that GHn values were positively correlated with the number of comorbidities per patient (GHn: Spearman’s ρ = 0.4,* P =* 0.004) while GHr and IGF-1 were not (*P* = 0.4 and* P* = 0.53, respectively). This data on GHn are confirmed in the ANOVA analysis (Fig. 1). When patients were divided into five groups based on the number of comorbidities, a trend of GHn to increase is confirmed (test for linear trend between means: F= 2.849, *P* = 0.035).

As expected, comorbidities were positively correlated with increasing age (Spearman’s ρ = 0.56, *P* < 0.0001).

**Figure 2:**
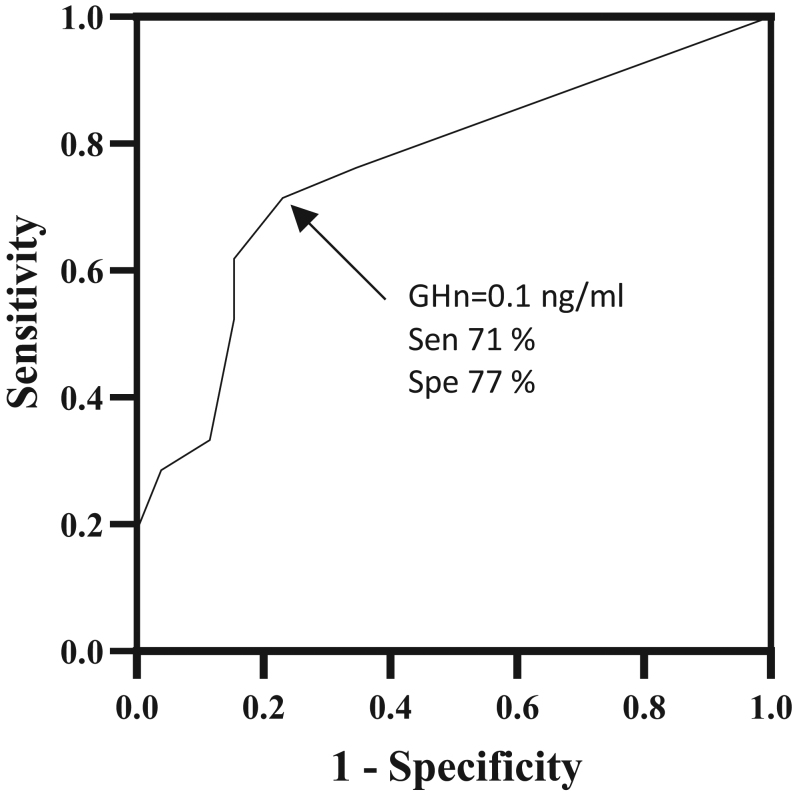
ROC curve of GH nadir levels for detecting the presence of a high rate of acromegalic comorbidities (at least three), AUC = 0.761, *P* = 0.002. The black square represents the GH nadir value of 0.1 ng/mL which showed the best combination of sensitivity and specificity for discriminating patients with and without a high rate of comorbidities (Sen 71% and Spe 77%). AUC, area under the curve; ROC, receiving operating characteristic; Sen, sensitivity; Spe, specificity.

We performed ROC analysis to evaluate the presence of a GHn value which could suggest the presence of a high rate of acromegaly complications (at least three comorbidities). In the whole group, the ROC curve (area under the curve (AUC) = 0.761) showed that the GHn cut-off of 0.1 μg/L (Youden’s J) pairs the highest sensitivity (71%) and specificity (77%) in the identification of a high rate of comorbidities (Fig. 2). In the whole group, the coexistence of acromegalic features and a GHn ≥ 0.1 μg/L were strongly associated with the presence of a high rate of comorbidities (at least three) with a sensitivity of 50% and specificity of 86% (OR of 6.0 with CI 1.421 to 22.61,* P =* 0.02).

### Follow up

The mean follow-up was 5.7 ± 3.4 years (median = 6 years). We registered an overall increase in the number of comorbidities per patient (median 3 vs 2,* P =* 0.002), especially in GR1 (GR1: median 4 vs 3, IQR 3–5,* P =* 0.04; GR2: 1 vs 1, IQR 0–3,* P =* 0.06). We observed an increase in some specific comorbidities: hypertension +18%, goitre +5.7%, overt DM +3.8%, malignant neoplasms +3.8%, cardiopathy +1.9%. Eight new colonoscopies were performed, resulting in +3.8% of colonic polyps (one polyp in a previous negative patient and one in a first screening). Carpal tunnel cases did not vary. Two patients with a previous malignancy were newly diagnosed with a different second neoplasm (one kidney and one lung cancer). We did not observe significant modifications in patients’ signs and symptoms of acromegaly.

**Figure 3:**
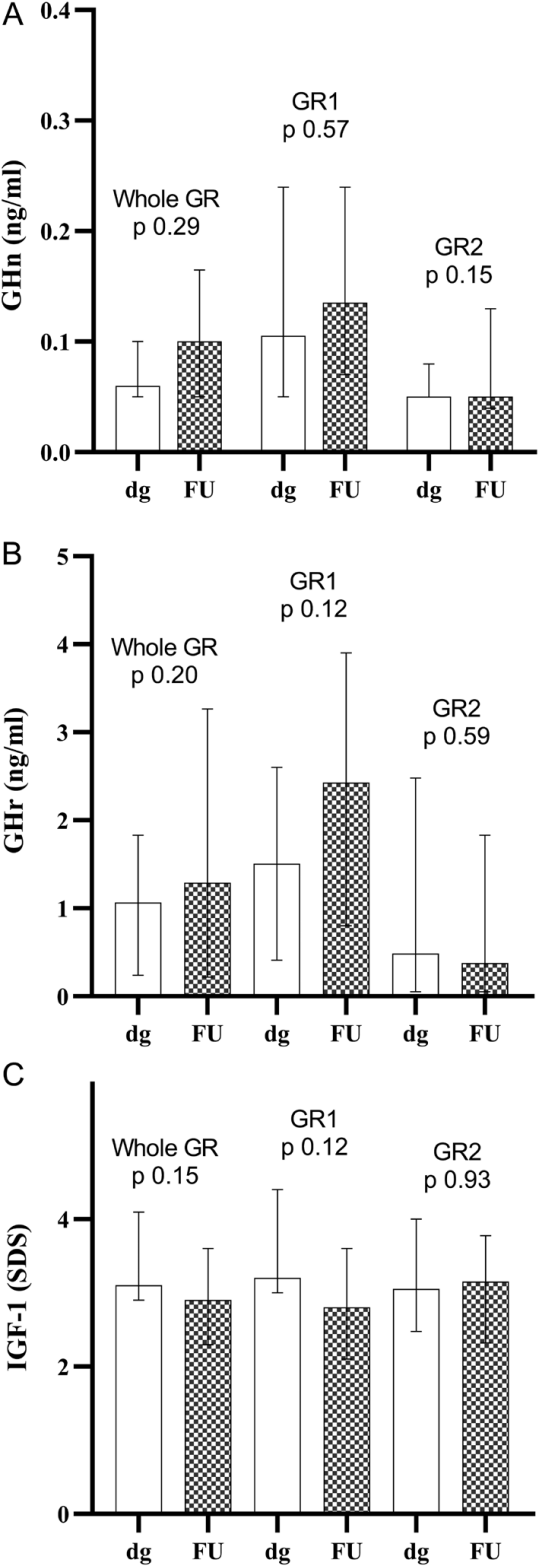
Hormonal evaluations at diagnosis (DG) and at the last available follow-up (FU). Mean FU time was 5.7 ± 3.4 years and we did not observe any significant modifications in GHn (A), GHr (B) and IGF-1 (C) values. These data confirm that our group of patients did not present acromegaly in its early stage but a 'low GH' acromegaly, characterized by stable GH and persistently high IGF-1 levels. Data are expressed as median with IQR range. GHn, GH nadir; GHr, GH random; dg, diagnosis; FU, follow-up; Whole GR, whole group of patients; GR1, group 1; GR2, group 2.

Interestingly, IGF-1 and GH levels did not significantly change, both in the whole group and subgroups (GR1: GHn +0.045,* P =* 0.570; GHr +1.58,* P =* 0.12; IGF-1 −0.72,* P =* 0.123. GR2: GHn + 0.02,* P =* 0.15; GHr +0.08,* P =* 0.594; IGF-1 +0.01,* P =* 0.93) (Fig. 3).

A neuroradiological follow-up was available in 31/45 patients with a previous MRI. We found five new microadenomas (three in GR1, two in GR2; maximum diameter range: 2.5–4 mm). The clinical, hormonal, and neuroradiological findings of all patients, are reported in the Supplementary Table (see Supplementary materials section provided at the end of the article).

### Treatment

One patient, presenting a macroadenoma and an ACTH-dependent hypercortisolism in addition to acromegalic features, was treated with TNS surgery. The histological report showed an ACTH+/GH+/LH−/FSH−/PRL−/TSH− immunostaining. After surgery, we observed a normalization of IGF-1 and cortisol levels. One more patient, with acromegalic features and no evidence of adenoma, was treated with somatostatin analogs (octreotide LAR 30 mg every 28 days) with good disease control. This patient previously underwent a Ga 68-DOTANOC PET imaging which resulted negative for ectopic hormonal secretion. According to our data showing a high rate of acromegalic comorbidities in GR1, other patients are currently under evaluation for a therapeutic option (medical treatment or surgery depending on the result of pituitary MRI).

## Discussion

In 2002, Barkan and coworkers introduced the term 'micromegaly' and, in the following years, other studies described similar patients characterized by acromegalic features, high IGF-1, but inappropriately low GH levels (7, 8, 9, 11, 14, 32, 33). Every study had a different design and aim, and GH nadir cut-off differed according to various guidelines, ranging from 2 μg/L to the most recent value of 0.4 μg/L. Therefore, it is likely that we would now define a lot of patients previously considered 'micromegalic' as acromegalic (7, 8, 9, 14). In the following paragraph, we describe the most relevant clinical observations collected in the literature.

In 2002, sixteen acromegalic patients showing a GHn <2 μg/L were reported, noting that 24-h mean GH values overlapped with healthy controls (14). Similarly, Ribeiro *et al.* in 2011, described seven acromegalic patients with GHn < 0.4 μg/L and a 'low 24-h GH profile' (17). All these patients had a histologically confirmed GH secreting pituitary microadenoma. In 2012, Subbarayan *et al.* confirmed the presence of few patients with a GHn < 0.4 μg/L in a group treated with TNS (9). Interestingly, in the study of Espinosa *et al.*, all acromegalic patients with GHn < 0.4 μg/L (three cases) showed a persistent disease after treatment (11).

Our study described a large cohort presenting with high IGF-1 and GHn < 0.4 μg/L. The group with acromegalic features, which we can also define as the 'micromegalic group', presented a higher prevalence of acromegalic comorbidities which is similar to the one reported in acromegaly (34, 35, 36, 37, 38, 39, 40, 41). Unfortunately, this is a retrospective study, and the number of diseases could be under-estimated, above all colonic polyps as few colonoscopies were performed. On the contrary, the age at diagnosis in this group was higher than expected in acromegaly (59 years vs 40–50 years) (42, 43, 44).

Follow-up data showed that both IGF-1 and GH levels remained stable while the prevalence of comorbidities significantly increased in the 'micromegalic group'. The main bias of this observation could be the expected health deterioration with aging, although the time of follow-up does not seem to be sufficient to justify such an increase. In our view, these data indicate that 'micromegaly' constitutes a distinct clinical entity, not being attributable to 'classic' acromegaly in its early stages.

Unlike previous studies showing a pituitary adenoma in almost all micromegalic patients, we found confirmatory imaging in only 57% (GR1). Nevertheless, this prevalence is higher than expected in the general population, especially for macroadenomas (9.5% vs 0.16–0.3%) (45, 46), suggesting the presence of a pituitary disorder. We also observed an increase in diagnoses of microadenomas during the follow-up period, speculating both on the improved MRI performance or on a real increase in adenoma’s volume. The low rate of macroadenomas in these patients, as previously described (7), confirms a different clinical presentation from classical acromegalic patients who mostly present with large lesions and only occasionally with negative MRI. The absence of a clear adenomatous area might represent a challenge for TNS surgery, as frequently faced in Cushing disease, but no large data on outcomes have been collected so far.

In our series, we described numerous patients with high IGF-1 levels despite no clear clinical suspicion of acromegaly (GR2). These patients were screened for GH hypersecretion mainly for the presence of another concomitant pituitary alteration. With respect to this group, IGF-1 evaluation harbors some limitations of its own as the lack of adequate age-adjusted normative data and susceptibility to interference from IGFBP. The lower incidence of acromegalic comorbidities in this group as well as in previous studies (9) supports the idea of an incidental finding with uncertain clinical significance. Likewise, higher GH levels in GR1 seem to support the presence of a GH-related disease in GR1 but not in GR2.

Various authors pointed out many uncertainties about the exact cut-off of GH nadir after OGTT, according to evidence on micromegalic patients. Some series also highlighted the need for individualization of reference values, according to gender, age, and BMI (31, 47). We could not confirm the presence of a GH-producing tumor in our series (except in one patient with an ACTH-GH co-hypersecretion) thus we were not able to associate a GHn cut-off value with the histological confirmation of disease. However, we identified 0.1 μg/L as the optimal cut-off to predict the presence of a high rate of acromegalic comorbidities in the setting of a condition previously identified as IGF-1/GH discrepancy. The combination of a GHn ≥ 0.1 μg/L and acromegalic features enhances the specificity in predicting the presence of multiple comorbidities (specificity = 86%). We, therefore, suggest a screening of acromegalic comorbidities in patients with these two characteristics.

Since GH hypersecretion has rarely an ectopic origin, we performed 68-Gallium imaging in one patient with a normal MRI and the exam resulted negative for abnormal uptakes (2). Considering that ectopic acromegaly is generally associated with very high GH levels (48), we decided not to undergo the other patients this expensive evaluation.

The pathophysiology of this 'low GH' acromegaly remains mostly unclear. It was pointed out by some authors that continuous exposure of the liver and other tissues to even minimally elevated tonic GH levels is enough to increase IGF-1 production into a supernormal range (12, 14). It is possible that these patients present a mildly high rate of GH production but tonic and sufficient to increase IGF-1 levels. A possible enhanced peripheral sensitivity to GH has also been postulated in some patients, for example in the presence of specific GH-receptor isoforms such as d3-GHr, which is proven to be associated with higher IGF-1 levels in patients treated with rGH (49, 50). Furthermore, with common GH assays, we mainly measure the 22-k circulating polypeptide, thus we cannot exclude the possible secretion of other active and not detected GH isoforms by the pituitary gland. However, no consistent evidence regarding all these hypotheses is available yet.

## Conclusions

In conclusion, patients with 'micromegaly', even in the presence of suppressed GH levels, present a high rate of comorbidities, showing no clinical differences from those with acromegaly. According to this evidence, we advise a revision of the GH nadir cut-off that, as already proposed, takes into consideration available assays and the patient’s characteristics (especially sex, estrogen status, and BMI). Considering the well-known impact of acromegalic complications on the quality of life and mortality, we suggest considering these patients as acromegalic, applying the same screening program to detect comorbidities and maintaining a close follow-up. Treatment should also be considered in selected patients. Additional data are needed in order to identify the precise pathophysiology of this 'low GH acromegaly' and to point out the best management.

## Supplementary Material

Supplementary data

## Declaration of interest

The authors declare that there is no conflict of interest that could be perceived as prejudicing the impartiality of this study.

## Funding

This work was supported by Ricerca Corrente funds from Fondazione IRCCS Ca’ Granda Ospedale Maggiore Policlinico and partially supported by grant NET-2018-12365454 from the Italian Ministry of Health and Regione Lombardia.
