# Supplementary material for: Clinical and hormonal findings in patients presenting with high IGF-1 and growth hormone suppression after oral glucose load: a retrospective cohort study
Source: Eur J Endocrinol. 2021 Jun 3;185(2):289–97. doi: 10.1530/EJE-21-0024 (PMC8284905; doi:10.1530/EJE-21-0024)
Supplement: Supplementary data [file supplementary_material.pdf]

**Supplementary data**

| | | | Diagnosis | | | Follow up | | | |
|---|---|---|---|---|---|---|---|---|---|
| Patient ID | Sex | Age (y) | IGF-1 (SDS) | GHn (μg/L) | MRI | IGF-1 (SDS) | GHn (μg/L) | MRI | FU time (y) |
| *Group 1* | | | | | | | | | |
| 3 | F | 59 | 3.1 | 0.23 | Micro | 2.40 | 0.14 | Micro | 4 |
| 4 | M | 72 | 3.0 | 0.05 | Normal | 2.50 | 0.05 | NA | 2 |
| 5 | F | 51 | 4.6 | 0.06 | Nonspecific alt. | 0.90 | 0.04 | NA | 6 |
| 7 | F | 58 | 2.7 | 0.12 | Micro | 2.10 | 0.25 | Micro | 7 |
| 12 | M | 55 | 3.3 | 0.31 | Micro | 4.60 | 0.22 | NA | 3 |
| 13 | F | 69 | 4.5 | 0.09 | Nonspecific alt | 2.10 | 0.15 | Micro | 3 |
| 14 | F | 65 | 3.2 | 0.05 | Macro | 4.60 | 0.36 | Macro | 9 |
| 16 | F | 80 | 2.7 | 0.3 | Micro | 1.40 | 0.40 | Micro | 7 |
| 18 | F | 72 | 3.3 | 0.12 | Nonspecific alt. | 2.90 | 0.15 | Micro | 11 |
| 19 | F | 57 | 6.7 | 0.33 | Normal | 3.30 | 0.10 | NA | 7 |
| 21 | F | 50 | 2.6 | 0.38 | Nonspecific alt. | 2.00 | 0.05 | Nonspecific alt. | 5 |
| 25 | F | 56 | 6.2 | 0.14 | Nonspecific alt. | 4.30 | 0.07 | Nonspecific alt. | 8 |
| 28 | F | 54 | 5.3 | 0.05 | Normal | 8.60 | 0.07 | Normal | 8 |
| 29 | F | 55 | 2.8 | 0.2 | Normal | 5.20 | 0.24 | Normal | 11 |
| 31 | M | 61 | 4.4 | 0.05 | ES | 2.90 | 0.24 | ES + micro | 10 |
| 32 | F | 59 | 2.5 | 0.09 | Macro | 3.80 | 0.09 | *TNS surgery* | 3 |
| 33 | F | 65 | 2.9 | 0.04 | Micro | 3.60 | 0.10 | Micro | 9 |
| 35 | M | 59 | 5.5 | 0.16 | Micro | 4.20 | NA | NA | 0.5 |
| 36 | F | 56 | 2.2 | 0.06 | NA | 1.60 | NA | NA | 0.5 |
| 38 | F | 69 | 4.2 | 0.13 | NA | 3.30 | 0.10 | NA | 0.5 |
| 39 | F | 50 | 2.1 | 0.12 | ES | 0.70 | 0.14 | NA | 0.5 |
| 41 | F | 69 | 3.4 | | Micro | 1.80 | NA | NA | 0.5 |
| 44 | F | 60 | 3.1 | 0.33 | Normal | 3.70 | 0.40 | Normal | 11 |
| 48 | F | 65 | 4.3 | 0.2 | NA | 2.40 | 0.06 | NA | 3 |
| 49 | M | 64 | 2.1 | 0.06 | NA | 1.10 | 0.15 | NA | 4 |
| *Tot.* | | *59* | *3.2* | *0.12* | | *2.9* | *0.14* | | *5* |
| *Group 2* | | | | | | | | | |
| 1 | F | 74 | 2.5 | 0.1 | Micro | 1.2 | 0.05 | Micro | 0.5 |
| 2 | F | 53 | 2.4 | 0.2 | Micro | 2.3 | 0.04 | NA | 11 |
| 6 | F | 20 | 3.1 | 0.27 | Normal | 3.8 | 0.17 | NA | 11 |
| 8 | M | 25 | 3.6 | 0.05 | Normal | 3.6 | 0.04 | NA | 3 |
| 9 | M | 54 | 2.3 | 0.04 | ES | NA | 0.08 | NA | 3 |
| 10 | F | 22 | 3.9 | 0.06 | ES | 2.7 | 0.05 | ES | 5 |
| 11 | M | 55 | 3.7 | 0.04 | NA | 6.2 | 0.23 | NA | 7 |
| 15 | M | 52 | 2.5 | 0.04 | ES | 1.9 | 0.04 | ES | 10 |
| 17 | M | 62 | 2.3 | 0.3 | Normal | 2.3 | 0.04 | Micro | 11 |
| 20 | F | 66 | 2.4 | 0.05 | Micro | 0.1 | | Micro | 8 |
| 22 | M | 24 | 2.7 | 0.04 | Normal | 3.1 | 0.05 | Micro | 10 |
| 23 | M | 39 | 3.4 | 0.05 | Normal | 2.2 | 0.05 | Normal | 7 |
| 24 | M | 72 | 4.1 | 0.05 | Micro | 3.9 | 0.05 | Normal | 6 |
| 26 | M | 38 | 2.2 | 0.04 | Macro | 2.7 | NA | Macro | 4 |
| 27 | M | 64 | 4.1 | 0.06 | Normal | 3.4 | 0.1 | NA | 8 |
| 30 | F | 20 | 2.0 | 0.06 | Micro | 3.5 | 0.13 | Micro | 8 |
| 34 | M | 41 | 3.7 | 0.04 | NA | 2.0 | 0.04 | NA | 0.5 |
| 37 | F | 21 | 3.1 | 0.05 | NA | 2.1 | NA | NA | 0.5 |
| 40 | F | 54 | 4.7 | 0.31 | NA | 3.2 | NA | NA | 0.5 |
| 42 | F | 52 | 5.4 | 0.27 | Macro | 3.8 | 0.1 | NA | 2 |
| 43 | M | 27 | 2.7 | 0.04 | ES | 3.4 | 0.04 | ES | 7 |
| 45 | M | 56 | 2.7 | 0.05 | Micro | 2.3 | 0.09 | Micro | 6 |
| 46 | M | 53 | 3.0 | 0.05 | Micro | 2.3 | 0.1 | Micro | 7 |

| | | | | | | | | | |
|---|---|---|---|---|---|---|---|---|---|
| 47 | F | 76 | 3.3 | 0.29 | ES e micro | 4.1 | 0.1 | ES + micro | 1 |
| 50 | F | 72 | 5.6 | 0.04 | Micro | 5.1 | 0.05 | Micro | 11 |
| 51 | F | 45 | 2.5 | 0.16 | ES | 2.3 | 0.16 | ES | 3 |
| 52 | F | 32 | 3.1 | 0.1 | Normal | 3.3 | 0.29 | Normal | 11 |
| 53 | F | 48 | 4.0 | 0.04 | Micro | 4.3 | NA | Micro | 3 |
| *Tot.* | | *52* | *3.1* | *0.05* | | *3.1* | *0.05* | | *6.5* |

**Supplementary table 1** Hormonal and neuroradiological data of patients included in the study (Group 1 and Group 2). M=male, F=female, SDS=standard deviation score, y=years, MRI=magnetic resonance imaging, FU=follow up, Micro=microadenoma, Macro=macroadenoma, Nonspecific alt.=nonspecific alterations, ES=empty sella, TNS=trans-nasal-sphenoidal surgery, NA=not available, Tot.=total (median values).
